# Natural capital accounting as a decision support tool for environmental management of a protected area in Madagascar

**DOI:** 10.1371/journal.pone.0321948

**Published:** 2025-05-09

**Authors:** Tony A. Ramihangihajason, Jean-Louis Weber, Solofo Rakotondraompiana, Edmond Roger, Miadana H. Faramalala, Solofoarisoa Rakotoniaina

**Affiliations:** 1 Institute and Observatory of Geophysics, Antananarivo (IOGA), University of Antananarivo, Antananarivo, Madagascar; 2 Department of Physics, Faculty of sciences. University of Antananarivo, Antananarivo, Madagascar; 3 International Consultant on Economic-Environmental Accounting, Former Senior Adviser to the European Environment Agency, Copenhague, Danemark,; 4 Ecole Supérieure Polytechnique d’Antananarivo, University of Antananarivo, Antananarivo, Madagascar; 5 Department of Plant Biology and Ecology, Faculty of Sciences, University of Antananarivo, Antananarivo, Madagascar; National Cheng Kung University, TAIWAN

## Abstract

Ecosystem change affects the availability of resources and services provided by nature. Ecosystem Natural capital accounting helps track these changes and supports better decision-making for managing the environment. This approach aims to assess changes in the stocks and flows of natural resources and the possibility to integrate them into economic and political decisions. The protected area of Mahavavy-Kinkony Complex, in North-Western of Madagascar, was chosen to implement this approach due to its many types of ecosystems as well as important reserves of threatened birds. In five years (2013–2018), we have observed a reduction in woodland cover (forest and mangrove) due to both regulated and illegal logging, linked to urban expansion and increasing of human pressure. This loss of woodland compromises not only biodiversity but also the capacity of ecosystems to provide ecosystem services. At the same time, the silting up of surface waters is compromising water quality and the health of aquatic ecosystems. In addition, the increase in agricultural land at the expense of forested areas raises concerns about the continuing degradation of natural ecosystems. All of these changes can be observed inside local socio-ecological landscape type. Each socio-ecological landscape type shows the potential variation in the production of ecosystem services.

## 1. Introduction

Awareness of the importance of ecosystems and concerns about their degradation due to human activities has become increasingly recurrent since the middle of 20th century. The first alarm was triggered by the creation of the Red List of Threatened Species by IUCN [1,2]. In 1972, the Club of Rome commissioned the Meadows Report on the Limits to Growth [3]. Then, the Stockholm conference in 1972 [4] highlighted this growing awareness and resulted in the creation of the United Nations Environment Programme (UNEP). The main idea put forward at this conference was to encourage ecologically sound management of the environment. But it was in the Brundtland report [5] that the notion of sustainable development emerged. The sustainable development paradigm underpinned the UN Conference on Environment and Development (UNCED), also known as the ‘Earth Summit’, held in Rio de Janeiro, in 1992 [6]. Outcomes of the Conference are the creation of the 3 conventions on climate change (UNFCCC), desertification (UNCCD) and biological diversity (UNCBD) and to the endorsement of the Agenda 21 [7]. Among other recommendations, the Agenda 21 called for the implementation of natural resource accounts in order to supplement the conventional National Accounts. It gave rise to the publication by UN Statistical Commission (1993) of the first manual of economic-environmental accounting, the so-called SEEA 93. The many experiments of the SEEA lead to the enlargement of its scope. In particular, the role of ecosystems and their services in sustainable development proposed first by Ehrlich and Mooney (1983) [8] was taken into account. The revision of the SEEA decided by the UN Statistical Commission in 2012 resulted in two volumes, the first one called System of Environmental Economic Accounting - Central Framework (SEEA-CF), the second one Ecosystem Accounting (SEEA-EA) [9]. While the SEEA-CF is framed by the classifications of industries and commodities of the System of National Accounts, the SEEA-EA is based on spatial mapping of ecosystems. During the SEEA revision process, the Secretariat of the CBD to fulfil the second objective of the strategic goal A in the Aichi Targets [10], published a technical report called: “Ecosystem Natural Capital Accounts: A Quick Start Package” (ENCA-QSP). It’s aims to implement Aichi Biodiversity Target 2 on Integration of Biodiversity Values in National Accounting Systems in the context of the SEEA Experimental Ecosystem Accounts [11]. While the SEEA-EA includes accounts in physical terms and monetary accounts, ENCA QSP focuses in only biophysical accounts. This initial version concerns only terrestrial ecosystems. In 2021 the SEEA-EA was adopted as an international statistical standard by the UN Statistical Commission [12] subject to monetary accounts being further validated. In the realm of biophysical accounts, SEEA EA and ENCA-QSP are broadly compatible.

Since then, a number of works have been carried out along two lines: implementation of accounts in biophysical terms and monetary valuations. Partial or comprehensive ecosystem accounts physical stocks and flows have been produced for a number of countries using international databases. Accounts targeting to monetary valuation of ecosystem services and assets are developed though case studies in programmes such as the Wealth Accounting and the Valuation of Ecosystem Services (WAVES program) [13] now known as the GPS [14], ARIES for SEEA (Artificial Intelligence for Environment & Sustainability) [15]; CARE-TDL or the Comprehensive Accounting in Respect of Ecology, Triple depreciation line [16]; INVEST or Integrated valuation of ecosystem services and trade-offs [17].

Environmental accounts have primarily been envisaged as an extension of the System of National Accounts, in order to go beyond the GDP (Gross Domestic Product) aggregate and adjust it from hidden environmental costs (negative externalities) or/and unrecorded benefits (positive externalities). In the evolution process of environmental accounting, it first appeared that accounts in biophysical terms were a prerequisite, needed prior to any monetary valuation. As well, the scale of accounting became of growing importance, both considering the analysis of environmental assets and the needs of users, would they be in charge of land management at various levels or companies willing to face their ecological liability.

The feasibility of nation-wide ecosystem accounting has been demonstrated [18–20], their interest as well as their limitations resulting from the use of international datasets. The challenge is now to develop accounts at smaller scales corresponding to more operational applications. In this study, we aim to show that ENCA can be applied at the local scale using high-resolution data and how they can be used as an effective environmental management tool.

In the first section of this paper, the ENCA approach will be summarised. Then, the study area will be described. After that, the results, land cover maps and the five ENCA accounts are presented. The interpretations of these accounts are given in order to obtain a large information useful for environmental managers. Discussions and conclusion will be the last part of this paper.

## 2. The ecosystem natural capital accounting (ENCA)

The following is mainly taking from Weber (2014) [11] which explain the ENCA terrestrial approach. The detailed methodology is available at this link: https://zenodo.org/records/14272568. The priority of ecosystem natural capital accounting (ENCA) is to show the ecological value of a given place and how ecosystems are evolving. The ecological value is a non-monetary assessment of integrity, health or resilience of ecosystems. All of which are important indicators for determining critical thresholds and minimum requirements to provide ecosystem services [21]. Over time, this ecological value of the study area can decrease, meaning the creation of ecological debts, or can increase meaning an accumulation of ecological credits.

ENCA ecosystem accounts are based on the land cover account which provides the spatial pattern common to other accounts. This is a diachronic analysis of land cover based on two Land Cover maps at the opening date and the closing date. Three ecosystems’ accounts are rooted on this Land Cover account: the ecosystem carbon account, the ecosystem water account and the ecosystem infrastructure integrity and services account. These accounts have the same structure: a set of four tables structured in the same way. The first one shows the basic balance sheet, which includes all ecosystem resource stocks and flows, natural and/or due to human activities between opening and closing dates. The second table measures the ecosystem potential resource and the amount accessible to human use due to constraints (e.g., nature protection). The third one contains all the uses of resources during the time intervals between opening and closing dates. The last table aims at calculating an sustainable use index (the ratio between accessible resources and used resources, compiled in previous parts) and an index of resilience or health of natural resources (a diagnostic based on variables considered as symptoms). The multiplication of sustainable use and health indexes is called ecosystem internal unit value. The average of the three indices of ecosystem internal unit value is then multiplied by the amount of accessible water resources, by the amount of accessible carbon resources and by the amount of accessible infrastructure functional services resources. These last values are the ecosystem capability unit (ECU) for each ecosystem type. Ecosystem capabilities has the status of ecological currency. Then the sum of all ecological values is the Total Ecosystem Capability (TEC) value. All calculation are performed inside analysis units called socio-ecological landscape units (SELUs) [22].

### 2.1 The socio-ecological landscape unit

Socio-ecological landscape units are areas within which all data are compiled and ecosystem accounts are calculated. SELUs integrate terrestrial and aquatic dimensions. Each SELUs is characterized by the dominant land cover and the boundaries of the sub-watershed.

### 2.2 The land cover account

The first step of producing the land cover account is to define all land cover classes. Weber (2014) [11] proposed the land cover system units (see Table 1) inspired from Di Gregorio *et al.* [23]. Sub-classes can be derived from as needed. This nomenclature is compatible with other nomenclature such as the Corine Land cover or others [24–26].

**Table 1:** The basic land cover ecosystem units.

Class ID	Land cover ecosystem units Class
1	Urban and associated developed area
2	Homogeneous herbaceous crop
3	Agriculture plantations, permanent crop
4	Agriculture associations and mosaics
5	Pastures and natural grassland
6	Forest tree cover
7	Shrubland, bushland, heathland
8	Sparsely vegetated area
9	Natural vegetation associations and mosaics
10	Barren land
11	Permanent snow and glaciers
12	Open wetlands
13	Inland water bodies
14	Coastal water bodies and inter-tidal areas

The dates of each image used in this study are for year 2013: 29 April for wet season and 03 August for dry season; for 2018 25 March for wet season, 04 October for dry season.

Each year’s images are actually made up of one Landsat image taken during the dry season and another Landsat image taken during the rainy season. Each Landsat 8 images include 11 spectral bands [27]. However, we only used the 6 following bands: the B2 (blue), B3 (green), B4 (red), B5 (near infrared), B6 (Short-Wave Infrared) and B7 (Short-Wave Infrared). All of these bands have a spatial resolution of 30m x 30m. To each of these spectral bands, we applied an image fusion algorithm [28] with B8 band (panchromatic) which has a spatial resolution of 15mx15m. The objective of image fusion step is to obtain synthetic images with 15mx15m spatial resolution but keeping the original spectral channels.

For each year, the two images (dry season image + rainy season image) are then stacked to form a single image with 15mx15m spatial resolution and 12 spectral bands.

Image of each year is classified using the supervised random forest method [29]. A supervised image classification method requires training and control pixels in a proportion of 70% for training and 30% to control the results. The confusion matrix gives the accuracy of classification.

The choice of training parcels depends on observations made during field work and the spectral responses of image. After classification made, hierarchical randomly points are chosen [30].

The Land Cover changes map can be derived directly from these two Land Cover maps [31], or can be obtained from the spatial image processing using a change detection algorithm to both images [32,33]. All the changes observed will be categorised into change flow classes according to their causes (see Table 2).

**Table 2:** The land cover flow classes.

Code	The land cover flows
lf1	Artificialisation
lf2	Extension of agriculture
lf3	Internal conversions and rotations
lf4	Management and alteration of forest areas
lf5	Habitat restoration and creation
lf6	Changes due to natural and multiple causes
lf7	Other changes in land not elsewhere classified and revaluation
lf0	No observed change in land cover

It is possible to have land cover flow sub-classes if necessary

### 2.3 The ecosystem water account

The water ecosystem account includes all continental waters and those of terrestrial ecosystems: water in rivers, canals, lakes and reservoirs, water in soil and vegetation, snow and glaciers. It also contains groundwater in its relationship with surface water. Exchanges with the atmosphere, such as precipitation and real evapotranspiration, are also taken into account. The same goes for water imported into the study area in form of beverage or other forms. User water system is connected to the resource system (canals and pipes, reservoirs) and wastewater returns are considered as secondary resources. In contrast, the account does not cover water stocks in the oceans and subsoil. It only records their relationship with terrestrial ecosystems and continental waters.

Databases on lakes and reservoirs can be used alone or combined with local data to obtain values of depth. For rivers, flows and lengths data also come from international database which may be adjusted with field data. For precipitation data, international database can also be used in combination with local data if available. The real evapotranspiration (ETr) values may be estimated from the precipitation (P) data, using a empirical formula:


ETr=(Pavg/ETravg)*P

ETr: real evapotranspiration

Pavg: Average Precipitation data

ETravg: average evapotranspiration

P: daily precipitation data

We made 380 depth measurement points along one profile over the Kinkony lake. One flow measurement was done on Mahavavy river. A household surveys were conducted on a sample of the population; the sample size is 320. The questionnaire concerns their use of natural resources including water, woods and timber and medicinal plants.

Water account is established for year 2013 and for year 2018.

Maps in Fig 5 show the observed changes in water between 2013 and 2018, The next Figure shows the maps of water changes between 2013 and 2018: stocks, accessible resources, total water use, use index and the water ecological internal unit value.

**Fig 1:**
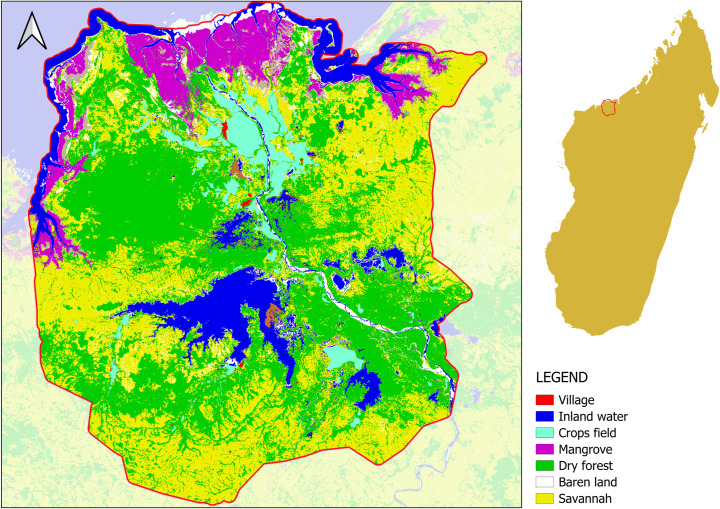
The protected area of Mahavavy-Kinkony Complex.

Average Precipitation data is from WorldClim database [34], the average evapotranspiration is from CGIAR database [35] and the daily precipitation data are from CHIRPS database [36].

### 2.4 The ecosystem carbon account

The ecosystem carbon accounts focus on terrestrial and aquatic ecosystems with biomass and soil organic carbon stocks and flows. Fossil carbon stocks are not part of this account. Emissions of greenhouse gas from biomass burning or processes generating methane and volatile organic compounds (VOCs) are already recorded in the basic balances.

This Ecosystem carbon account records the stocks and flows of organic carbon available in soil, in underground and above-ground vegetation, and in water (fish and plant species). The gross primary production (GPP) flow of biomass by natural and cultivated vegetation is considered as a carbon flux.

The main data used in this account are the (i) above ground biomass that is obtained using data from local and/or regional ecological surveys and allometric equation [37] and the land cover maps. Since no data on underground carbon is generally available, underground carbon is assumed to show no change; (ii) Soil organic carbon values are, for example, from ISRIC Soil Grids with 250 m ground resolution [38]; (iii) GPP may be taken from MODIS website [39]; and (iv) household surveys provide information on quantity of resources used by local population. The Ecosystem Infrastructure functional services account.

### 2.5 The ecosystem landscape infrastructure functional services account

Infrastructure functional services are also called intangible services. ENCA assumes that an ecosystem in good condition continuously provides ecosystem services.

Therefore, ENCA uses landscape indicators to assess the potential of ecosystems to provide intangible ecosystem services.

Three fundamentals’ landscapes indices are used:

- The Green Landscape Background Index (GBLI) which represents the Land Cover biomass potential;
- The High Natural Value Index (HNVI) takes into account the level of protection of an area;
- The Landscape Fragmentation Index (FI) shows the level of fragmentation of the ecosystem. It is considered a negative effect.

The fragmentation index as proposed by Werber (2014) [11] is obtained using the Effective Mesh Size [40]. But 2 more indices are added in our study because in general there are very few roads and many paths. Inspired from McGarigal (1995) [41], the two new indices, the numbers of patches and surface ratio between tree cover and the river basin area, are added. To obtained a final fragmentation index, the average of these three indices are calculated.

The multiplication of these three indices (GBLI, HNVI, FI) gives the Landscape Ecosystem Potential or LEP [18].

For rivers, three other indices are also calculated:

- The River Accessibility Weighted Index is a measurement of a potential based on river extent (their length) and discharge (for each river section, length x log (discharge value));
- HNVI-river which is the result of intersection of HNVI with the rasterized rivers;
- River fragmentation which is the number of dams in each watershed.

The composite index of river potential is obtained by multiplying the last three indices. The index is then superposed with SELUs map. One obtains the mean index value for each SELU. This is then multiplied by the area of each SELU to obtain the River Ecosystem Potential (REP).

The Total Ecosystem Potential (TEIP) of the ecosystem infrastructure is the sum of the LEP and REP values.

### 2.6 The ecosystem capital capability account

The three ecosystem accounts each generate indices representing the unit ecological value of each ecosystem type. This is the result of multiplying the usage index by the ecosystem health index. When these values are then multiplied by the corresponding available resources, these indices collectively determine the overall ecosystem capacity, known as the ecosystem capability of the area. [18]

The capability of an ecosystem means its overall potential to deliver all services in a sustainable way without reducing the potential for other services. The difference between the capability values at the closing and opening dates is called the ecological debt, if negative, or ecological credit if positive. Ecological debt reflects the degree of degradation of ecosystems and ecological credit reflects the degree of improvement of the ecosystem states.

### 2.7 Study area

The Mahavavy-Kinkony Complex is a protected area located between 15°46’ and 16°12’ South latitude and 45°27’ and 45°56’ East longitude in North-western of Madagascar (see Fig 1). It covers some 350,000 ha. The northern part of the site opens onto the Mozambique Channel. The elevation is varying from 0 to 150 m [42]. It is a category V protected area according to the International Union for Conservation of Nature (IUCN) classification. Its status as protected area is obtained in 2015 [43]. The protected area includes several villages and two small cities. The landscape is composed of various ecosystems: forests (dry forest and mangrove), savannah, lakes, marine and coastal ecosystems. Mahavavy-Kinkony Complex is a refuge for endemic and threatened terrestrial and aquatic species found in different natural habitats such as lakes, rivers, swamps, mangroves, and dry forests. At the mouth of the Mahavavy River, the Mahavavy Delta is the largest area of mangroves [42]. The mangrove extends over 80 km along the coast. The Kinkony lake is the second largest one in Madagascar, and a number of threatened waterbird species can also be 250 seen here [42].

The region’s climate is a dry tropical climate with two contrasting seasons, dry season from April to October and rainy season from November to March. The total of annual rainfall is 1554 mm, with a maximum in January (475.6mm) and a minimum in June (0.6mm). The average annual temperature is 26°C, with a minimum of 18°C in July and a maximum of 35°C in December [42].

For the application of ENCA to the Mahavavy-Kinkony Complex protected area, the years of 2013 and 2018 have been selected as opening and closing dates. The choice of these two dates is due to the fact that the protected area of Mahavavy-Kinkony Complex was established in 2015 [36]. This allows for a comparison of the state of the ecosystem before the protection was established and the state of ecosystems after the establishment of the protected area status.

## 3. Results

Each ecosystem accounts are full presented on this section and the maps of some indicators. All maps were produced based on research results and generated using QGIS.

### 3.1 The land cover account

Table 3 shows land cover classes in the Mahavavy-Kinkony Complex protected area, and how they were derived from the primary land cover classes as proposed by Weber (2014).

**Table 3:** Nomenclature adopted for the Mahavavy-Kinkony Complex protected area.

Class ID	Basic Class (land cover ecosystem units)	Sub-class ID	Sub-class used for mahavavy-Kinkony Complex’s LC	Description
1	Urban and associated developed area	11	Village	Town infrastructure grouping (dense and less dense such as hamlets)
2	Homogeneous herbaceous crop	21	Rice field	The specialized agricultural practice of growing rice
3	Agriculture plantations, permanent crop	31	Sugar-cane field	Plantation dedicated to the production of sugar cane
4	Agriculture associations and mosaics	41	Crops field	Field made up of various and undistinguished crops
5	Pastures and natural grassland	51	Savannah	Field made up of herbaceous cover and also Shrubs like Bismarkia nobilis at very low density (<10%)
6	Forest tree cover	61	Dry forest	Dry deciduous forest with more than 70% tree cover
		62	Spares dry forest	Dry deciduous forest made up of trees between 30% and 70%
		63	Dense mangrove	Mangrove greater than 70% tree cover.
		64	Scattered mangrove	Mangrove less than 70% tree cover.
		65	Stunted mangrove	Mangrove regeneration
7	Shrubland, bushland, heathland	71	Shrubland	Herbaceous cover and shrubs
10	Barren land	101	Baren land	Natural land not covered by vegetation
		102	Tan	Geological formation that extends beyond the mangrove, sometimes conducive to the formation of herbaceous vegetation
12	Open wetlands	121	Phragmites	Herbaceous plants of varying density on Kinkony lakes (bird habitat)
		122	Raffia	Area covered by raffia which is also a water reservoir
13	Inland water bodies	131	Water bodies (lakes…)	Inland water

On the left columns we find the classes as proposed by Weber (2014) with the ID of each class. On the two middle columns, we have the ID and the name of each subclass. The right column gives the description of each subclass. The ID of a subclass is derived from the ID of parent class by adding digit.

**Table 4:** The Mahavavy-Kinkony Complex land cover account.

Land cover classes →	Village	Rice field	Sugar-cane field	Raffia	Crops field	Savannah	Dense dry forest	Dry forest with density less than 70%	Dense mangrove	Scattered mangrove	Stunted mangrove	Tan	Shrubland	Baren land	Phragmites	Water bodies	Total(ha)
Land cover stocks and flows ↓																	
**Opening stock 2013**	**433**	**4 256**	**4 728**	**959**	**5 817**	**63 937**	**50 031**	**42 800**	**6 816**	**14 482**	**3 047**	**6 530**	**82 737**	**8 391**	**2 284**	**53 514**	**350 762**
F_lf1 Artificial development	119																119
F_lf2 Agricultural extension		2 529	881	40	8 692	1 857										7	14 006
F_lf3 Internal conversions		11	228	–	26	–	7 899	12 855	303	5 227	878	521					27 948
F_lf4 Management and alteration of forest land						18 129							16 898	298			35 325
F_lf5 Restoration and development of habitats						165	–	1 837	1	292	77	1 333	12 028	3			15 736
F_lf6 Changes of land-cover due to natural and multiple causes														721	212	4 833	5 768
F_lf7 other land cover changes																	–
**Total formation of land cover**	**119**	**2 540**	**1 109**	**40**	**8 718**	**20 151**	**7 899**	**14 692**	**304**	**5 519**	**955**	**1 854**	**28 926**	**1 024**	**212**	**4 844**	**98 902**
C_lf1 Artificial development						63		38					17	1			119
C_lf2 Agricultural extension						2 010	774	2 778	–	146	–	120	6 136	1 198	548	296	14 006
C_lf3 Internal conversions				37	228		7 805	6 255	2 189	802	1 716	369	8 547				27 948
C_lf4 Management and alteration of forest land							2 848	16 487	–	1 844	14	2 748	11 384				35 325
C_lf5 Restoration and development of habitats				155	16	13 807								1 096	320	342	15 736
C_lf6 Changes of land-cover due to natural and multiple causes				28	2	377		18	1	236	53	198	648	3 183	226	798	5 768
C_lf7 other land cover changes																	
**Total consumption of land cover**	**–**	**–**	**–**	**220**	**246**	**16 257**	**11 427**	**25 576**	**2 190**	**3 028**	**1 783**	**3 435**	**26 732**	**5 478**	**1 094**	**1 436**	**98 902**
Net change in land cover = F - C	119	2 540	1 109	-180	8 472	3 894	-3 528	-10 884	-1 886	2 491	-828	-1 581	2 194	-4 454	-882	3 408	–
**Closing Stock 2018**	**552**	**6 796**	**5 837**	**779**	**14 289**	**67 831**	**46 503**	**31 916**	**4 930**	**16 973**	**2 219**	**4 949**	**84 931**	**3 937**	**1 402**	**56 922**	**350 762**

F_ means formation area of the specified Land cover between 2013 and 2018 and C_ means consumption area of the specified Land cover for the same period. Values are expressed in ha.

The following Table 3 presents the different entities present in the land use maps. This nomenclature is derived from the original nomenclature proposed by Weber (2014) and which is compatible with other nomenclatures.

**Fig 2:**
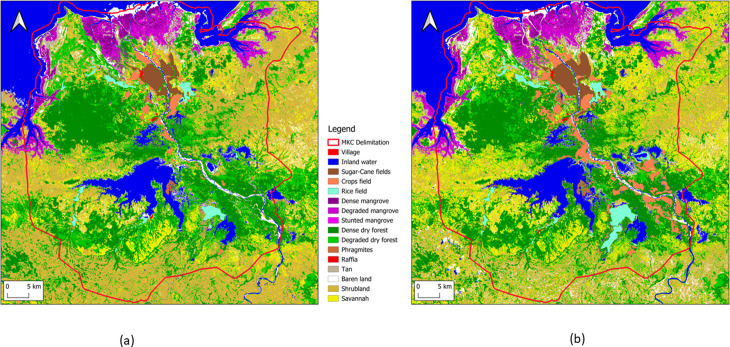
Mahavavy-Kinkony Complex Land cover maps. (a) maps in 2013 and in (b) in 2018.

The following Fig 2 shows the two land cover maps of the year 2013 and the year 2018.

**Fig 3:**
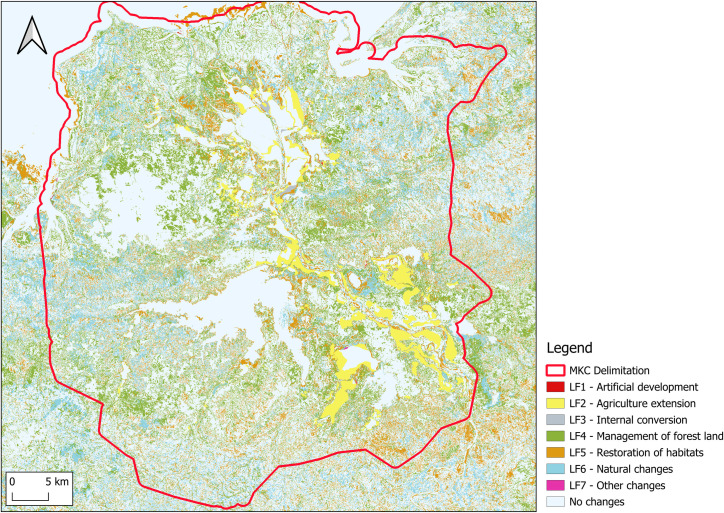
Mahavavy-Kinkony Complex land cover flow.

The next Fig 3 shows the map of land cover changes between 2013 and 2018. It is obtained by taking the difference between the two previous maps.

The following Table 4 presents the diachronic analysis of land use, spanning both 2013 and 2018.

### 3.2 Socio-ecological landscape units

**Fig 4:**
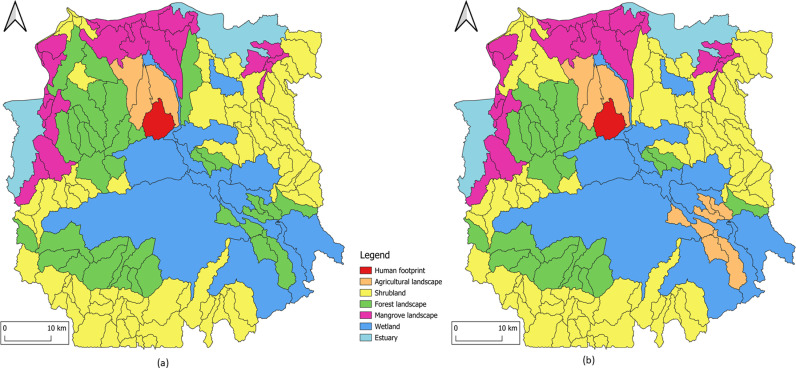
Mahavavy-Kinkony Complex maps of the SELU. (a) in 2013 and (b) in 2018.

The next Fig 4 shows the maps of the SELU in 2013 and in 2018.

Changes in DLT (dominant land cover type) alert in areas under fast, such as on the North-East and South-West of the maps Fig 4.

### 3.3 The ecosystem water account

**Fig 5:**
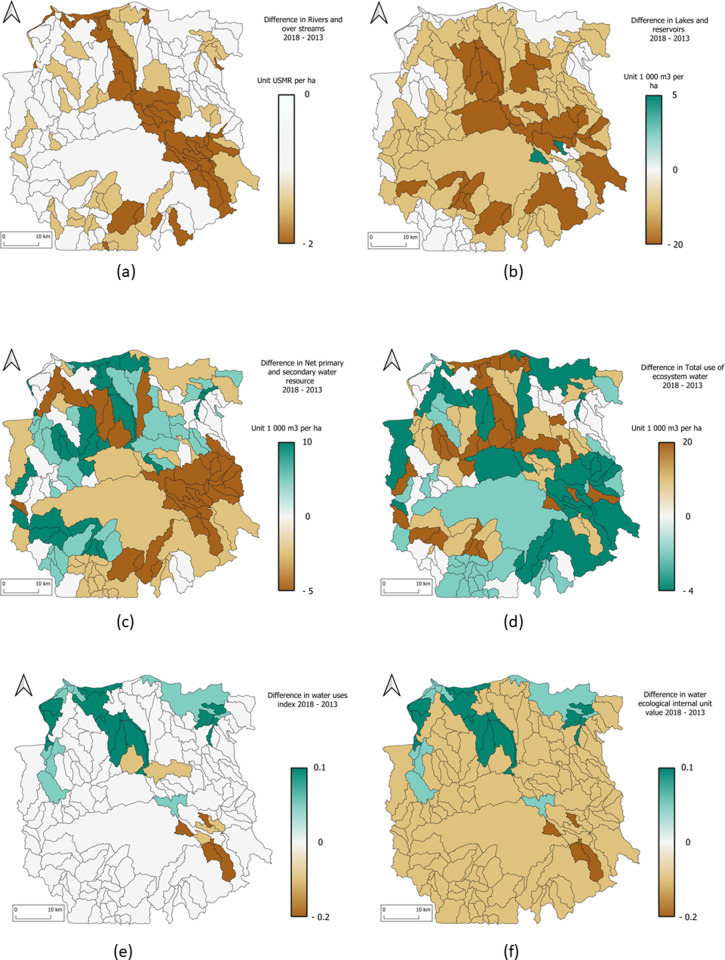
Mahavavy-Kinkony Complex maps of changes per unit area (ha). (a) in drainage (b) lakes and reservoirs (c) accessible water resource (d) total of uses (the positive value shows that the uses of water resources are increasing between 2013 and 2018), (e) water use index and (f) water internal value.

The Fig 5 shows the changes map of all important indices in water account.

The Tables 5 and 6 shows the ecosystem water account of Mahavavy-Kinkony Complex protected area in 2013 and 2018.

**Table 5:** The ecosystem water account of protected area in 2013.

Socio-ecological landscape unit →		Human footprint	Agricultural landscape	Shrubland	Forest landscape	Mangrove Landscape	Wetland	Estuary	Total
Codes	Long names								
W1_1	Lakes and reservoirs	21 902	631 953	65 950	53 272	245 213	1 881 588	232 905	3 132 783
W1_2	Rivers and over Streams (SRMU)	1 560	91 233	19 085	52 114	34 641	33 945	101	232 678
W1_4	Ground water	11 924	52 085	447 648	125 536	384 629	40 479	21 999	1 084 299
W1_5	Soil and vegetation	4 169	16 436	10 240	44 797	63 078	11 399	3 910	154 029
W2_1	Precipitation	13 556	60 836	123 516	148 094	106 165	44 977	23 556	520 699
W2_2	Groundwater drainage to river minus percolation	-52 918	-190 448	-1 177 985	-213 204	-2 155 697	-114 477	-274 299	-4 179 028
W2_3	Natural inflows from upstream territories	54 880	267 173	1 264 727	297 375	2 983 773	187 558	381 044	5 436 530
W2_4	Artificial inflows of water from other territories and the sea	3 359	10 077	54 257	17 204	1 944	11 645	41	98 527
W2_5	Waste water returns/discharge to inland water assets	5	23	26	18	7	4	0	84
W2_6	Other returns of abstracted water to inland water bodies	7 832	23 487	126 536	40 122	4 463	27 167	93	229 699
W2	Total inflows of water = SUM (W21 to W26)	26 712	171 148	391 077	289 609	940 655	156 875	130 435	2 106 511
W3_1	Actual evapotranspiration	13 261	51 540	85 930	145 897	47 569	47 015	18 649	409 860
W3_3	Natural outflows to downstream territories and the sea	2 256	86 021	124 327	86 368	886 672	71 044	111 653	1 368 340
W3_4	Abstraction from inland water bodies	35 872	107 710	57 930	18 371	21 405	124 263	464	366 015
W3_8	Artificial outflow of water to other territories and the sea	–	38	1 181	78	2 223	27	98	3 644
W3	Total outflows of water = SUM (W34 to W39)	73 757	312 375	341 656	365 314	970 505	319 959	131 124	2 514 690
W4	Net ecosystem water balance = W2-W3	-47 050	-141 251	49 394	-75 723	-29 856	-163 089	-689	-408 263
W4a	Available effective rainfall = W2_1 - W3_1	295	9 296	37 586	2 197	58 596	-2 038	4 908	110 839
W2a	Total natural renewable water resources (TNWR) = W21 + W22 + W23	15 517	137 560	210 257	232 265	934 241	118 059	130 302	1 778 201
W2b	Total secondary water resources = W24 + W25 + W26	11 195	33 587	180 819	57 345	6 414	38 816	134	328 310
W6	Net primary & secondary water resource = W2a+W2b-W32-W33	24 456	85 127	266 750	203 241	53 983	85 831	18 782	738 171
W9	Total Use of Ecosystem Water = W3_1 + W3_4	49 133	159 249	143 861	164 267	68 974	171 278	19 112	502 033
W13	Index of water use	0.50	0.53	1.00	1.00	0.78	0.50	0.98	0.76
W14	Composite index of ecosystem water health	0.95	0.95	0.95	0.95	0.95	0.89	0.95	0.95
W15	Water ecological internal unit value	0.72	0.74	0.98	0.98	0.87	0.70	0.97	0.85

The unit is 1000 m^3^

**Table 6:** The ecosystem water account of Mahavavy-Kinkony Complex protected area in 2018.

	Socio-ecological landscape unit →	Human footprint	Agricultural landscape	Shrubland	Forest landscape	Mangrove Landscape	Wetland	Estuary	Total
ENCA codes	Long Names								
W1_1	Lakes and reservoirs	8 690	460 471	27 159	19 364	210 324	1 746 585	46 205	2 518 799
W1_2	Rivers and over Streams (SRMU)	1 547	131 401	21 510	11 479	31 093	33 671	100	230 801
W1_4	Ground water	11 454	77 640	465 890	93 036	333 535	38 884	21 132	1 041 571
W1_5	Soil and vegetation	2 363	14 224	63 305	20 996	31 830	6 618	2 235	141 571
W2_1	Precipitation	7 149	49 191	119 260	146 731	103 916	43 151	21 629	491 026
W2_2	Groundwater drainage to river minus percolation	- 41 746	-243 909	-2 477 019	- 68 268	-1 052 898	- 88 201	-210 773	-4 182 814
W2_3	Natural inflows from upstream territories	43 550	320 972	2 569 369	151 721	1 780 934	148 836	302 376	5 317 757
W2_4	Artificial inflows of water from other territories and the sea	3 910	20 746	44 245	12 305	2 965	9 902	38	94 111
W2_5	Waste water returns/discharge to inland water assets	4	21	24	14	4	3	0	71
W2_6	Other returns of abstracted water to inland water bodies	9 118	48 376	103 158	28 691	6 842	23 099	84	219 368
W2	Total inflows of water = SUM (W21 to W26)	21 985	195 397	359 036	271 193	841 764	136 790	113 354	1 939 519
W3_1	Actual evapotranspiration	6 589	41 979	89 845	144 942	52 164	41 758	15 748	393 024
W3_3	Natural outflows to downstream territories and the sea	2 364	84 275	121 765	85 241	779 787	62 028	97 484	1 232 946
W3_4	Abstraction from inland water bodies	52 132	138 291	58 984	16 404	39 454	132 018	498	437 781
W3_8	Artificial outflow of water to other territories and the sea	–	65	1 409	3	2 191	28	120	3 817
W3	Total outflows of water = SUM (W34 to W39)	61 084	264 610	272 003	246 590	873 597	235 832	113 851	2 067 567
W4	Net ecosystem water balance = W2-W3	- 30 147	57 041	298 643	254 786	800 119	4 744	112 736	1 497 922
W4a	Available effective rainfall = W2_1 - W3_1	560	7 212	29 415	1 789	51 751	1 393	5 881	98 002
W2a	Total natural renewable water resources (TNWR) = W21 + W22 + W23	8 952	126 254	211 610	230 183	831 952	103 786	113 232	1 625 969
W2b	Total secondary water resources = W24 + W25 + W26	13 033	69 143	147 426	41 010	9 812	33 004	122	313 550
W6	Net primary & secondary water resource = W2a+W2b-W32-W33	19 621	111 122	237 271	185 952	61 976	74 761	15 870	706 574
W9	Total Use of Ecosystem Water = W3_1 + W3_4	58 720	180 269	148 829	161 346	91 618	173 776	16 246	830 805
W13	Index of water use	0.33	0.62	1.00	1.00	0.68	0.43	0.98	0.72
W14	Composite index of ecosystem water health	0.95	0.95	0.95	0.95	0.95	0.87	0.95	0.95
W15	Water ecological internal unit value	0.64	0.78	0.98	0.98	0.81	0.65	0.96	0.83

The unit is 1000 m^3^

### 3.4 The ecosystem carbon accounts

**Fig 6:**
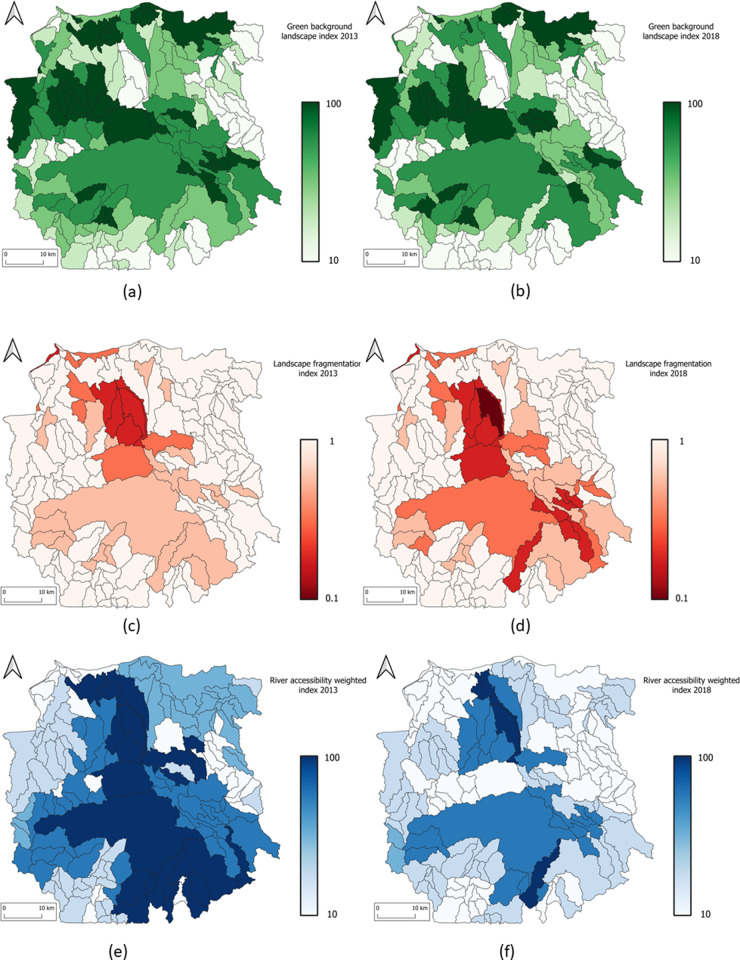
Mahavavy-Kinkony Complex maps of ecosystem carbon account. (a) opening stock of carbon for 2013 and (b) for 2018, (c) the changes of carbon accessible resources, (d) the variation of total use of carbon (the positive values shows that the use of carbon resources is increasing in 2018 compared to 2013), the sustainable use index for (e) 2013 and (f) for 2018.

The ecosystem carbon account is calculated for year 2013 and for year 2018. The Fig 6 shows the maps of opening stocks of carbon, the flows of the ecosystem carbon, the changes of total use of carbon, and the sustainable use index for 2013 and for 2018.

Tables 7 and 8 shows the ecosystem carbon account of mahavavy-Kinkony Complex protected area in 2013 and 2018.

**Table 7:** The ecosystem carbon account of Mkcmahavavy-Kinkony Complex protected area in 2013.

	Socio-ecological landscape unit →	Human footprint	Agricultural landscape	Shrubland	Forest landscape	Mangrove Landscape	Wetland	Estuary	Total
ENCA codes	Long names								
C1_1	Aboveground living biomass carbon	223 553	1 057 753	1 708 762	8 247 555	7 739 968	712 260	327 633	20 017 485
C1_2	Litter and deadwood carbon	26 826	126 930	205 051	989 707	928 796	85 471	39 316	2 402 098
C1_31	Roots carbon	133 014	582 026	944 209	3 937 828	4 015 635	379 369	177 264	10 169 345
C1_32	Soil Organic Carbon	6 134	32 721	223 141	68 518	251 363	20 425	12 243	614 545
C1_4	Livestock carbon	94	418	2 909	944	2 203	388	151	7 107
C1	Opening Stocks Total	389 621	1 799 849	3 084 073	13 244 553	12 937 965	1 197 914	556 606	33 210 581
C2_3	NPP (Net Primary Production)	207 658	891 086	1 493 705	5 715 808	4 593 957	607 149	262 628	13 771 990
C2	Total inflow of biocarbon (total gains)	402 358	1 764 273	3 288 089	11 087 464	9 457 152	1 205 660	546 297	27 751 293
C3_11	Cereal	913	1 676	325	171	52	1 615	7	4 759
C3_13	Sugar canes	1 362	18 643						20 005
C3_199	Other crops	32	132	16	10	4	76		269
C3_3	Livestock grazing	18 050	65 424	492 409	197 351	283 440	46 877	18 632	1 122 183
C3_4	Roundwood net removals	29 601	128 003	695 661	302 581	494 052	91 227	25 831	1 766 956
C3	Total withdrawals of biocarbon	57 305	244 115	1 257 263	582 424	830 325	164 390	46 681	3 182 504
C4_33	Combustion of wood fuel roundwood	3 442	16 576	41 142	14 174	48 623	3 514	1 934	129 406
C4_34	Combustion of other biocarbon fuel	881	3 746	8 231	9 758	6 306	2 926	265	32 114
C4	Net indirect anthropogenic losses of biocarbon	4 323	20 322	49 373	23 932	54 930	6 441	2 199	161 520
C5	Total use and induced loss of ecosystem biocarbon	61 628	264 437	1 306 637	606 356	885 255	170 831	48 880	3 344 024
C10_1	Net accessible biomass carbon inflow	198 885	873 072	2 236 962	5 052 486	4 703 786	601 319	282 179	13 948 690
C10_2	Index of limitations of use due to nature protection	0.70	0.70	0.81	0.10	0.10	0.73	0.70	0.48
C10	NEACS_Net Ecosystem Accessible Carbon Surplus	139 219	611 151	1 792 252	505 249	470 379	434 790	197 526	4 150 565
**SCU**	**Sustainable intensity of carbon use index (C10/C5)**	**1.00**	**1.00**	**1.00**	**0.83**	**0.53**	**1.00**	**1.00**	**0.91**
CEH	Ecosystem Carbon Health Index	1.00	1.00	1.00	1.00	1.00	1.00	1.00	1.00
**CIUV**	**Ecosystem Carbon Internal Unit Value**	**1.00**	**1.00**	**1.00**	**0.92**	**0.77**	**1.00**	**1.00**	**0.95**

The unit is ton of Carbon

**Table 8:** The ecosystem carbon account of Mahavavy-Kinkony Complex protected area in 2018.

	Socio-ecological landscape unit →	Human footprint	Agricultural landscape	Shrubland	Forest landscape	Mangrove Landscape	Wetland	Estuary	Total
ENCA codes	Long names								
C1_1	Aboveground living biomass carbon	218 319	1 639 711	1 035 681	7 189 968	6 679 512	668 015	317 357	17 748 562
C1_2	Litter and deadwood carbon	26 198	196 765	124 282	939 335	801 541	80 162	38 083	2 206 366
C1_31	Roots carbon	132 078	876 054	562 797	3 710 963	3 323 126	353 744	167 367	9 126 130
C1_32	Soil Organic Carbon	6 268	48 613	253 675	53 078	232 497	20 902	12 449	627 482
C1_4	Livestock carbon	95	731	3 086	665	1 921	397	147	7 042
C1	Opening Stocks Total	382 959	2 761 873	1 979 521	11 894 009	11 038 598	1 123 219	535 404	29 715 582
C2_3	NPP (Net Primary Production)	191 660	1 335 774	930 326	5 376 135	4 423 786	595 022	252 775	13 105 478
C2	Total inflow of biocarbon (total gains)	393 299	2 779 237	2 282 224	10 525 031	8 931 563	1 236 664	519 903	26 667 922
C3_11	Cereals	1 046	1 991	1 727	135	56	3 224	11	8 191
C3_13	Sugar canes	1 596	24 062						25 658
C3_199	Other crops	61	321	21	16	4	288		712
C3_3	Livestock grazing	17 371	101 076	542 225	161 617	292 962	45 690	20 263	1 181 204
C3_4	Roundwood net removals	61 137	395 911	954 505	409 015	449 492	171 400	27 660	2 469 119
C3	Total withdrawals of biocarbon	121 539	793 848	1 853 105	809 645	847 758	338 729	52 974	4 817 599
C4_33	Combustion of wood fuel roundwood	3 588	19 248	47 493	11 974	46 142	3 457	2 327	134 229
C4_34	Combustion of other biocarbon fuel	4 840	32 582	42 481	28 545	12 625	14 047	605	135 726
C4	Net indirect anthropogenic losses of biocarbon	8 428	51 831	89 974	40 519	58 767	17 504	2 932	269 955
C5	Total use and induced loss of ecosystem biocarbon	129 967	845 679	1 943 079	850 164	906 526	356 233	55 906	5 087 553
C10_1	Net accessible biomass carbon inflow	206 075	1 445 065	1 900 312	4 818 784	4 409 565	645 816	268 364	13 693 982
C10_2	Index of limitations of use due to nature protection	0.70	0.72	0.80	0.10	0.10	0.73	0.70	0.52
C10	NEACS_Net Ecosystem Accessible Carbon Surplus	144 253	1 028 159	1 495 989	481 878	440 957	466 831	187 855	4 245 922
**SCU**	**Sustainable intensity of carbon use index**	**1.00**	**1.00**	**0.77**	**0.57**	**0.49**	**1.00**	**1.00**	**0.83**
CEH	Ecosystem Carbon Health Index	1.00	1.00	1.00	1.00	1.00	1.00	1.00	1.00
**CIUV**	**Ecosystem Carbon Internal Unit Value**	**1.00**	**1.00**	**0.88**	**0.78**	**0.74**	**1.00**	**1.00**	**0.92**

The unit is ton of Carbon

### 3.5 The ecosystem infrastructure functional services account

**Fig 7:**
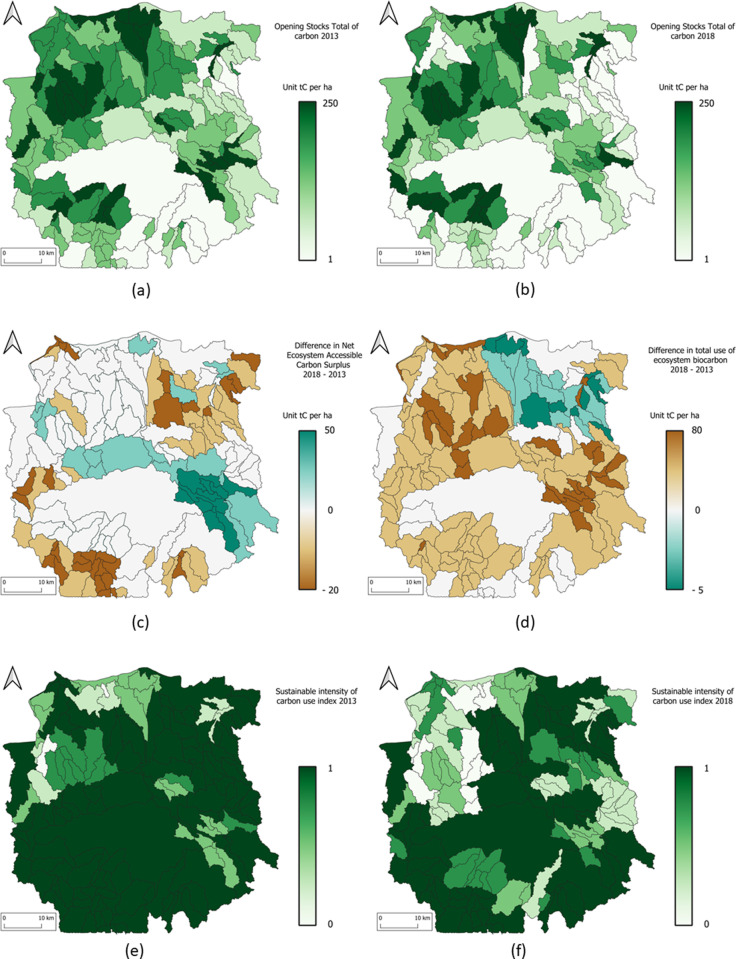
Mahavavy-Kinkony Complex Maps of three ecosystem infrastructure functional services indices. Green Background Landscape Index in (a) 2013 and in (b) 2018, Fragmentation Index in (c) 2013and in (d) 2018, River Accessibility Weighted Index in (e) 2013 and in (f) 2018.

The Fig 7 shows three indices in ecosystem infrastructure functional services.

The Table 9 shows the ecosystem infrastructure functional services account of mahavavy-Kinkony Complex protected area between 2013 and 2018.

**Table 9:** The ecosystem infrastructure functional services account of Mahavavy-Kinkony Complex protected area.

	Socio-ecological landscape unit →	Human footprint	Agricultural landscape	Shrubland	Forest landscape	Mangrove Landscape	Wetland	Estuary
ENCA codes	Long names							
RC1	Opening stock of land cover	6 241	17 408	121 514	79 706	39 927	110 957	21 397
2013_GBLI	Green background landscape index (GBLI) (average per pixel)	59.91	59.36	69.53	88.01	81.75	79.10	86.23
HNVI	Landscape high nature conservation value index	1.00	1.00	1.00	1.14	1.00	1.05	1.00
IF_13	Landscape fragmentation index	0.62	0.55	0.97	0.98	0.98	0.96	1.00
LEP13	LEP composite index	37.15	32.65	67.35	98.63	79.97	80.16	86.17
	Pixel size in ha (15m)	0.0225	0.0225	0.0225	0.0225	0.0225	0.0225	0.0225
NLEP13	Net Landscape Ecosystem Potential	5 216	12 788	184 136	176 876	71 846	200 129	41 486
RAWI_2013	River accessibility weighted index	53.38	75.38	15.43	18.77	21.69	33.46	13.80
HNVI_riv	River high nature conservation index	1.00	1.00	1.00	1.14	1.00	1.05	1.00
frag_riv_20	River fragmentation	1.00	1.00	1.00	1.00	1.00	1.00	1.00
REP13	REP composite index	53.38	75.38	15.46	21.41	21.69	35.28	13.80
NREP13	Net river ecosystem potential	7 496	29 524	42 256	38 396	19 489	88 088	6 644
TEIP1_13	Opening stock of Total ecosystem infrastructure potential	12 713	42 312	226 391	215 273	91 334	288 217	48 130
RC2	Closing stock of land cover	6 241	29 596	129 468	60 649	38 842	110 957	21 397
2018_GBLI	Green background landscape index (GBLI) (average per pixel)	59.37	65.68	68.83	88.17	80.35	76.62	86.52
HNVI	Landscape high nature conservation value index	1.00	1.00	1.00	1.19	1.00	1.05	1.00
IF_18	Landscape fragmentation index	0.52	0.47	0.97	0.97	0.97	0.87	1.00
LEP18	LEP composite index	30.91	30.86	66.71	102.02	77.61	69.89	86.44
NLEP18	Net Landscape Ecosystem Potential	4 341	20 556	194 324	139 212	67 824	174 479	41 613
RAWI_2018	River accessibility weighted index	15.01	37.71	9.44	11.32	13.18	16.53	8.98
HNVI_riv	River high nature conservation index	1.00	1.00	1.00	1.19	1.00	1.05	1.00
frag_riv_20	River fragmentation	1.00	1.00	1.00	1.00	1.00	1.00	1.00
REP_18	REP composite index	15.01	37.71	9.46	13.47	13.18	17.43	8.98
NREP18	Net river ecosystem potential	2 108	25 115	27 547	18 377	11 523	43 510	4 324
TEIP2_18	Closing stock of Total ecosystem infrastructure potential	6 449	45 671	221 871	157 589	79 347	217 990	45 937
TEIP1_adj	adjustment	14 349	47 757	255 525	242 975	103 088	325 306	54 324
TEIP2_adj	adjustment	7 000	49 573	240 828	171 053	86 127	236 615	49 862
**EISU**	**Ecosystem infrastructure use intensity**	**0.49**	**1.00**	**0.94**	**0.70**	**0.84**	**0.73**	**0.92**
EHI_EcoHea	Composite ecosystem health index	0.94	0.94	0.92	0.92	0.96	0.93	0.94
**EIIUV**	**Annual change in ecological internal unit value**	**0.71**	**0.97**	**0.93**	**0.81**	**0.90**	**0.83**	**0.93**

### 3.6 The ecosystem capability account

**Fig 8:**
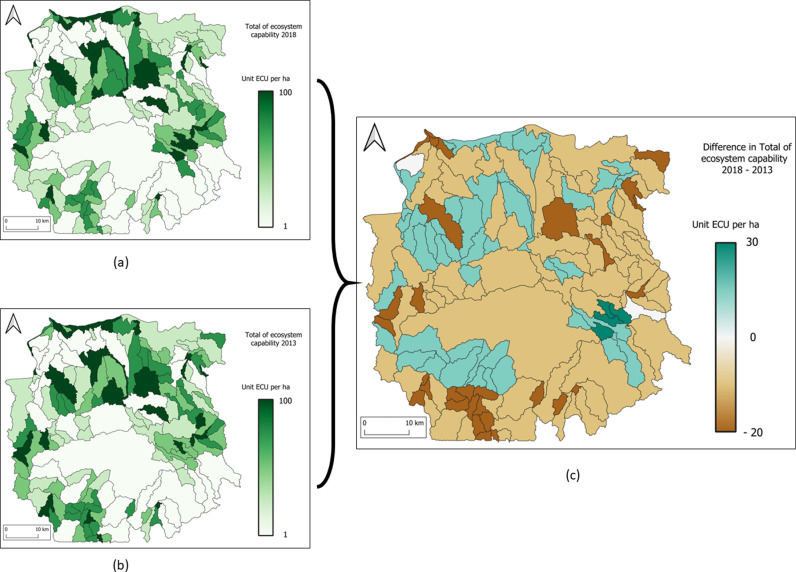
Mahavavy-Kinkony Complex maps of total ecosystem capabilities. (a) 2013 and (b) 2018, (c) the trend in five years.

For this account, three maps are show to illustrate the total ecosystem capabilities (see Fig 8) of Mahavavy-Kinkony Complex protected area and the trend of Total Ecosystem Capability in 5 years.

The Tables 10 and 11 shows the ecosystem capability account of mahavavy-Kinkony Complex protected area in 2013 and in 2018. The Table 12 show the difference in ecosystem capability between 2013 and 2018.

**Table 10:** The ecosystem capabilities account of Mahavavy-Kinkony Complex protected area in 2013.

	Socio-ecological landscape unit →	Human footprint	Agricultural landscape	Shrubland	Forest landscape	Mangrove Landscape	Wetland	Estuary	Total
C10	NEACS_Net Ecosystem Accessible Carbon Surplus	139 219	611 150	1 792 252	505 248	470 378	434 790	197 525	
CIUV	Ecosystem Carbon Internal Unit Value	1.00	1.00	1.00	0.92	0.77	1.00	1.00	
W6	Net primary and secondary water resource	24 456	85 126	266 749	203 241	53 983	85 831	18 782	
W15	Water ecological internal unit value	0.72	0.74	0.98	0.98	0.87	0.70	0.97	
TEIP1	Opening stock of Total ecosystem infrastructure potential	14 348	47 757	255 524	242 975	103 087	325 306	54 324	
EIIUV	Annual change in ecological internal unit value	0.71	0.97	0.93	0.81	0.90	0.83	0.93	
ECU_P	ECU average [by SELU] = (CIUV+WIUV+EIIUV)/3	0.81	0.90	0.97	0.90	0.84	0.84	0.97	
C_EC_13	Carbon Ecosystem Capability	113 027	552 392	1 736 426	455 338	396 642	365 739	190 670	3 810 235
W_EC_13	Water Ecosystem Capability	19 855	76 942	258 441	183 164	45 521	72 200	18 130	674 254
EI_EC_13	Ecosystem Infrastructure Capability	11 649	42 981	247 566	218 973	86 928	273 643	52 439	934 179
TEC_13	TOTAL ECOSYSTEM CAPABILITY (ECU)	144 532	672 315	2 242 432	857 476	529 091	711 581	261 240	5 418 668

**Table 11:** The ecosystem capabilities account of Mahavavy-Kinkony Complex protected area in 2018.

2018	Socio-ecological landscape unit →	Human footprint	Agricultural landscape	Shrubland	Forest landscape	Mangrove Landscape	Wetland	Estuary	Total
C10	NEACS_Net Ecosystem Accessible Carbon Surplus	144 252	1 028 158	1 495 989	481 878	440 956	466 831	187 854	
CIUV	Ecosystem Carbon Internal Unit Value	1.00	1.00	0.88	0.78	0.74	1.00	1.00	
W6	Net primary and secondary water resource	19 621	111 121	237 271	185 951	61 976	74 761	15 869	
W15	Water ecological internal unit value	0.64	0.78	0.98	0.98	0.81	0.65	0.96	
TEIP2	Closing stock of Total ecosystem infrastructure potential	7 000	49 573	240 827	171 053	86 126	236 614	49 862	
EIIUV	Annual change in ecological internal unit value	0.71	0.97	0.93	0.81	0.90	0.83	0.93	
ECU_P	ECU average [by SELU] = (CIUV+WIUV+EIIUV)/3	0.78	0.91	0.93	0.87	0.81	0.82	0.96	
C_EC_18	Carbon Ecosystem Capability	111 871	931 272	1 396 558	418 890	359 365	382 281	181 138	3 781 374
W_EC_18	Water Ecosystem Capability	15 217	100 651	221 501	161 645	50 508	61 221	15 302	626 045
EI_EC_18	Ecosystem Infrastructure Capability	5 429	45 111	224 821	148 694	70 190	193 760	48 079	736 085
TEC_18	TOTAL ECOSYSTEM CAPABILITY (ECU)	132 516	1 077 034	1 842 880	729 229	480 063	637 262	244 520	5 143 504

**Table 12:** The difference between ecosystem capability in 2013 and 2018.

Socio-ecological landscape unit →	Human footprint	Agricultural landscape	Shrubland	Forest landscape	Mangrove Landscape	Wetland	Estuary	Total
TEC 2018-TEC 2013	-12 016	404 719	-399 553	-128 247	-49 028	-74 319	-16 720	-275 164
Percentage	-8	61	-18	-15	-9	-10	-6	-5
Ecological debt (ECU)	-275 164							

## 4. Discussions

Analysing the results of ecosystem natural capital accounting makes it possible to assess changes in the quantity and health of ecosystems. the discussion aims to clarify the information and indices derived from the accounting process, highlighting their implications for environmental sustainability, policy decisions and natural resource management.

### 4.1 The land cover account

The land cover account is shown in Table 4. The area of villages has increased by 119 ha (+27%) between 2013 and 2018. This trend can be considered as a proxy for population growth as the huts generally have the similar dimensions. According to the United Nations Population Fund [44] annual population growth in Madagascar is + 2.8% (+14% in five years). For mahavavy-Kinkony Complex this rate has almost doubled. The existence of migration is therefore justified. mahavavy-Kinkony Complex managers estimate a migration rate of + 2.5% [42]. These last values give a population growth of + 26.5% in 5 years.

For rice cultivation, an increase of 2,540 hectares (+60%) was recorded over five years. Most of this conversion came from natural land cover (vegetation including forest). Before the migration phenomenon, the main economic activities of local population are fishing (Kinkony lake and at sea), and working in the sugar factory of Namakia city. The new migrants settle in the south-east of lake Kinkony and cultivate rice and food crops. These people certainly come from the neighbouring regions of Marovoay and Ambato-Boeny, other rice growing areas. These migrants are no longer true climate migrants [45], as rice cultivation requires financial investments and relatively long time before harvest.

The establishment of 1,109 hectares (+26%) of sugarcane plantations over five years is linked to the revival of the sugarcane factory in the small city of Namakia.

The pressure of human activities has reduced the raffia area by 180 hectares (-19%) in five years. Raffia tree only grows in area with water. Reduction of raffia area means decrease of water resource. One cause of this phenomena is the creation of rice fields Regarding cultivated fields, the population is converting savannah and forests cover into agricultural land, resulting in the creation of 8,692 hectares (+146%) from natural cover (Savannah and forest). This is similar to the trend in rice cultivation. Savannah areas have increased, with a total gain of 3,894 hectares (+6%). The creation of 18,129 hectares (line F_LF4) is due to the disappearance of forest, mangroves, and shrubs. The consumption (disappearance) of 13,807 hectares of savannah (line C_LF5) is attributed to forest and shrubland restoration efforts [42] Forest cover decreased by 3,528 hectares due to selective logging for construction purposes. For sparse dry forests, the loss of 10,884 hectares (-25%) is mainly caused by illegal charcoal production [42], primarily intended to the major city of Majunga. Additionally, the collection of medicinal plants, hunting, and sacred rituals are common practices among villagers. These activities lead to the creation of paths within forests, increasing accessibility and contributing to forest degradation. This explains the significant degradation of sparse forests, amounting to 12,855 hectares.

The net total change in dense, degraded, and stunted mangroves is -223 hectares. Generally, this reduction is due to selective wood cutting for construction. However, sedimentation also impacts wetland areas by altering soil structure [46]. The sedimentation phenomenon is evidenced by the increase of 721 hectares of bare soil and sand, which affects mangroves.

The destruction of Phragmites in Lake Kinkony (-882 hectares or -38%) is linked to the creation of rice fields in the Phragmites marsh areas and uncontrolled fires in the region. This threat is exacerbated by natural events such as cyclones, which disperse Phragmites away from the lake during the dry season. The plants are either burned or perish on land. Destruction of Phragmites leads to biodiversity loss and water quality degradation. It is the natural habitat of birdlife, and also provides a suitable area for fish [47,48].

### 4.2 The ecosystem water account

The general trend (Tables 5 and 6) indicates a decrease in available water over five years (-20%). For lakes and reservoirs (line W1_1), one cause of this decline is sedimentation, which was already noted by Randriamasimanana and Rabarimanana (2011) [46]. For rivers (line W1_2), the difference between 2013 and 2018 is linked to reduced flow rates, which in turn are connected to decreased of precipitation. Similar phenomenon is observed in the total water inflow, comprising precipitation and natural inflows from upstream watersheds.

However, in agriculture, water use has increased by about +43% compared to 2013 level. This is due to the creation of new agricultural sites. This has led to changes in some SELUs.

Total water outflow includes evapotranspiration, anthropogenic use, and natural outflow downstream. The latter is generally tied to flow rates, as it partly reflects runoff from the area. The reduction (-17%) in total water outflow between 2013 (2 514 690) and 2018 (2 067 567) was observed.

The values of sustainability use index are below 1 except for savannahs and forests. According to Fig 5E, a small improvement (+0.1) in the situation can be seen in the north of the CMK, where sugar cane is grown. Accessible water resources in the area have increased, hence this improvement (see Table 6 line W6). The situation has not therefore improved naturally, but by accumulating more water to compensate for the increased demand.

Water quality has remained relatively stable compared to the 2018 situation.

### 4.3 The ecosystem carbon accounts

The general trend indicates a decrease in carbon over five years. Each SELU (socio-ecological landscape unit) includes some degree of forest cover, even if it is not dominant. The exploitation of this forest cover explains the observed trend in each SELU. Although other types of land cover exist, carbon is more concentrated in trees. However, wood harvesting has increased over the five-year period (see Tables 7- and 8-line C3). The total values of carbon uses are 3,182,504 for 2013 and 4,817,599 for 2018. It reflects overexploitation of wood, as these figures are abnormally high compared to population growth. In line C3, the extraction within the agricultural landscape SELU nearly tripled over five years, driven by the conversion of natural land to arable land (see land cover account Table 4).

According to carbon account lines C3_11 and C3_199 (see Tables 7 and 8), the values indicate an increase in agricultural production. It remains insufficient because production methods are still rudimentary.

For four out of the six SELUs, uses of carbon resource (SCU) are sustainable, as the use index are still equal to 1, except for savannah, which is scored 0.77 in 2018. This is due to population growth leading to an increase in demand of Bismarck palm. However, forests and mangroves have scores of 0.83 and 0.53 respectively in 2013, and 0.57 and 0.49 in 2018. These values indicate overexploitation of forests and mangroves. The main activities contributing to this are charcoal production and logging for construction. These activities are quite widespread although illegal and despite restrictions.

### 4.4 The ecosystem infrastructure functional services account

For the Green Background Landscape Index, the forest and mangrove SELUs show the highest values (see Table 9 and Fig 7A and 7B). For river accessibility water index, the highest values are found in urban, agricultural, and wetland SELUs (see Fig 7E and 7F). However, due to declining of river flow rates (as shown in the water account – Tables 5 and 6), these values have also decreased.

Since the hard-core boundaries of the protected area have not changed, the HNVI indicator remains the same over five years. Regarding landscape fragmentation, urban and wetland SELUs are the most fragmented. Agricultural SELUs show a decrease of fragmentation index value. This is because agricultural area is more uniform. Fragmentation index values for forests and mangroves remain stable.

Overall, EISU indicator (Table 9) shows a downward trend, except for agricultural SELUs. This is due to the conversion of some SELUs (including forest) into agricultural ones,

For human footprint SELUs, EISU = 0.49. This decrease is linked to the reduction of river accessibility weighted index (Table 9). This situation can be a harbinger of water supply issue in urban areas.

### 4.5 The ecosystem capability account

The creation of agricultural areas has led to an increase in the production of ecosystem services in these zones (increase of + 61% of Total Ecosystem Capability – TEC – see Table 12). However, this increase is primarily related to provisioning services, while other types of services are not represented. This is evidenced by the low value GBLI (global background landscape index) of these agricultural areas compared to others (see Table 9).

Over five years, the protected area experienced a -5% reduction in its capacity to provide all ecosystem services (see Table 12). This decline corresponds to an ecological debt of -275,164 ECU. This reduction is not solely linked to any specific natural resources but can be attributed to the simultaneous decline of multiple resources, highlighting the interconnections between different types of natural resources. In addition to reforestation programmes, it is recommended to address other phenomena, such as the silting-up of water surfaces inside the protected area.

### 4.6 Limits of the study

While the landscape units approach used in this study provides a valuable framework for linking land cover dynamics to broader land systems science and archetype literature, it has inherent limitations that should be acknowledged. One key limitation arises from the methodology employed to categorize and assess landscape units based on the dominant land cover. The use of dominant land cover can obscure significant changes within the landscape, especially in cases where a shift in land cover occurs but remains within the same landscape unit.

This limitation becomes particularly important when considering ecological processes within these landscape units. Such transformations can affect biodiversity, ecosystem services, and land management strategies, but these changes may not be accurately captured if only the dominant land cover is considered. Thus, while this methodology is a good starting point for analyzing land cover dynamics on a large scale, it may oversimplify landscape changes and overlook some important consequences.

One limitation of this study is the selection of only two specific years, 2013 and 2018, as the opening and closing dates for the application of the ENCA to the Mahavavy-Kinkony Complex protected area. While these years provide a useful comparison of the ecosystem state before and after the establishment of the protected area in 2015, they may not fully capture the dynamics and ongoing changes within the ecosystem over time. Additionally, the absence of data prior to 2013 limits the ability to assess long-term trends and pre-existing conditions of the area before the protected status was even considered. This narrower timeframe may overlook potential shifts in the ecosystem that occurred before or immediately after the establishment of the protection status, which could provide a more comprehensive understanding of the ecological impacts of the protected area.

On the other hand, the overall accuracy of the maps, expressed as a percentage (see appendix), is an indicator of their reliability. For 2013, the overall accuracy of 90% indicates that the land cover maps correctly identify the cover categories in 90% of cases. For 2018, an overall accuracy of 91% is slightly higher than in 2013, and indicates a decline in the reliability of land cover maps. Although these levels of accuracy are high, they warn of the need to continue refining classification methods and data processing to achieve an even more accurate representation of land landscapes.

Also, In-situ measurement data, obtained directly in the field, provides detailed and accurate information that reflects the real and specific conditions at each site. However, these data have limitations, such as their geographical representativeness. They are often limited to areas where observations and sampling have been carried out. International data from global sources can cover vast geographical areas and provide comparisons between different regions and ecosystems. However, these data also have their limitations, such as spatial resolution. International data may be insufficient to identify fine detail and heterogeneity within local ecosystems.

This study is exclusively focused on the mahavavy-Kinkony Complex protected area without taking into consideration interactions with the surrounding buffer zones. These interactions can affect trophic relationships and food webs. Buffer zones can also play a key role in ecological resilience by providing refuges or alternative resources in case of disturbances. However, it should be noted that buffer zones are generally difficult to access.

## 5. Conclusion

Our study on ecosystem natural capital account of the mahavavy-Kinkony Complex’s protected area revealed how ecosystem is evolving. It allows managers to make well-adapted decisions.

Results of ENCA analysis can be now shown as maps which are more easily understandable. ENCA recommends the valuation of ecological debts and credits on the basis of restoration costs. Ecological balance-sheets would be then important tool for integrating biodiversity into financial risks assessments. They would complement the carbon balances presently in use by financial or environmental institutions for climate change with much needed comprehensive ecological vision. Financial institutions such as Banque de France [49].are working for such enlargement. Similar approach is the proposed by Vardon et al (2021) [50]. They proposed the use of benchmark for an environmental bank. The value of ENCA ecological debt/credit can be used for that.

## Supporting information

S1 Appendix The confusion matrix of 2013 classification result. (DOCX)
