# Supplementary material for: Natural capital accounting as a decision support tool for environmental management of a protected area in Madagascar
Source: PLoS One. 2025 May 9;20(5):e0321948. doi: 10.1371/journal.pone.0321948 (PMC12064043; doi:10.1371/journal.pone.0321948)
Supplement: S1 Appendix — (DOCX) [file pone.0321948.s001.docx]

# Appendix

## The confusion matrix of 2013 classification result

|  |  | Control pixels | | | | | | | | | | | | | | | |  |
| --- | --- | --- | --- | --- | --- | --- | --- | --- | --- | --- | --- | --- | --- | --- | --- | --- | --- | --- |
| 2013 |  | Village | Rice field | Sugar-cane field | Raffia | Crops field | Grassland | Dense dry forest | Dry forest with density less than 70 % | Dense mangrove | Scattered mangrove | Stunted mangrove | Tan | Shrubland | Baren land | Phragmites | Water bodies | Total (%) |
| Classified image | Village | 0.12 | - | - | - | - | - | - | - | - | - | - | - | - | - | - | - | 0.12 |
|  | Rice field | - | 1.21 | - | - | - | - | - | - | - | - | - | - | - | - | - | - | 1.21 |
|  | Sugar-cane field | - | - | 1.30 | - | 0.05 | - | - | - | - | - | - | - | - | - | - | - | 1.35 |
|  | Raffia | - | - | - | 0.25 | - | 0.01 | 0.01 | 0.01 | - | - | - | - | 0.01 | - | - | - | 0.27 |
|  | Crops field | - | - | - | - | 1.66 | - | - | - | - | - | - | - | - | - | - | - | 1.66 |
|  | Grassland | - | - | - | - | - | 16.74 | - | 0.37 | - | - | - | 0.74 | 0.37 | - | - | - | 18.23 |
|  | Dense dry forest | - | - | - | 0.29 | - | - | 13.39 | 0.58 | - | - | - | - | - | - | - | - | 14.26 |
|  | Dry forest with density less than 70 % | - | - | - | 0.46 | - | 0.46 | 0.46 | 10.13 | - | 0.23 | - | - | 0.46 | - | - | - | 12.20 |
|  | Dense mangrove | - | - | - | - | - | - | - | - | 1.80 | 0.10 | 0.05 | - | - | - | - | - | 1.94 |
|  | Scattered mangrove |  | - | - | - | - | - | - | - | 0.08 | 3.80 | 0.17 | - | - | - | 0.08 | - | 4.13 |
|  | Stunted mangrove | - | - | - | - | - | - | - | - | 0.02 | - | 0.85 | - | - | - | - | - | 0.87 |
|  | Tan | - | - | - | - | - | - | - | - | - | - | - | 1.81 | - | 0.05 | - | - | 1.86 |
|  | Shrubland | - | - | 0.44 | 0.44 | - | 0.87 | 0.44 | 0.87 | - | - | - | 0.44 | 20.09 | - | - | - | 23.59 |
|  | Baren land | - | - | 0.08 | - | - | - | - | - | - | - | - | - | - | 2.31 | - | - | 2.39 |
|  | Phragmites | - | 0.02 | - | - | - | - | - | - | - | - | - | - | - | 0.02 | 0.61 | - | 0.65 |
|  | Water bodies | - | - | - | - | 0.46 | - | - | - | - | 0.46 | - | - | - | - | 0.46 | 13.87 | 15.26 |
|  | Total (%) | 0.12 | 1.24 | 1.82 | 1.43 | 2.17 | 18.08 | 14.30 | 11.97 | 1.91 | 4.59 | 1.06 | 2.99 | 20.93 | 2.38 | 1.15 | 13.87 | 100.00 |
| Overall Accuracy = 90 % | |  |  |  |  |  |  |  |  |  |  |  |  |  |  |  |  |  |

## The confusion matrix of 2018 classification result

|  |  | Control pixels | | | | | | | | | | | | | | | |  |
| --- | --- | --- | --- | --- | --- | --- | --- | --- | --- | --- | --- | --- | --- | --- | --- | --- | --- | --- |
|  |  | Village | Rice field | Sugar-cane field | Raffia | Crops field | Grassland | Dense dry forest | Dry forest with density less than 70 % | Dense mangrove | Scattered mangrove | Stunted mangrove | Tan | Shrubland | Baren land | Phragmites | Water bodies | Total (%) |
| Classified image | Village | 0.16 | - | - | - | - | - | - | - | - | - | - | - | - | - | - | - | 0.16 |
|  | Rice field | - | 1.74 | - | - | - | - | - | - | - | - | - | - | - | - | - | - | 1.74 |
|  | Sugar-cane field | - | - | 1.66 | - | - | - | - | - | - | - | - | - | - | - | - | - | 1.66 |
|  | Raffia | - | - | - | 0.22 | - | - | 0.01 | - | - | - | - | - | - | - | - | - | 0.22 |
|  | Crops field | - | 0.14 | - | - | 3.94 | - | - | - | - | - | - | - | - | - | - | - | 4.07 |
|  | Grassland | - | - | - | - | 0.37 | 17.48 | 0.37 | 0.37 | - | - | - | - | 0.37 | - | 0.37 | - | 19.34 |
|  | Dense dry forest | - | - | - | 0.28 | - | - | 12.71 | 0.28 | - | - | - | - | - | - | - | - | 13.26 |
|  | Dry forest with density less than 70 % | - | - | - | 0.18 | - | 0.18 | 0.18 | 8.21 | - | - | - | - | 0.36 | - | - | - | 9.10 |
|  | Dense mangrove | - | - | - | - | - | - | - | - | 1.33 | 0.04 | 0.04 | - | - | - | - | - | 1.41 |
|  | Scattered mangrove |  | - | - | - | - | - | - | - | 0.19 | 4.27 | 0.09 | - | - | - | 0.09 | - | 4.65 |
|  | Stunted mangrove | - | - | - | - | - | - | - | - | 0.02 | 0.02 | 0.60 | - | - | - | - | - | 0.63 |
|  | Tan | - | - | - | - | - | - | - | - | - | - | - | 1.38 | - | 0.04 | - | - | 1.42 |
|  | Shrubland | - | - | 0.43 | 0.43 | - | 0.87 | 0.43 | 0.87 | - | 0.43 | - | - | 20.32 | - | 0.43 | - | 24.21 |
|  | Baren land | - | - | - | - | - | - | - | - | - | - | - | 0.06 | - | 1.06 | - | - | 1.12 |
|  | Phragmites | - | 0.02 | - | - | - | - | - | - | - | - | - | - | - | - | 0.39 | - | 0.41 |
|  | Water bodies | - | - | - | - | - | - | - | - | - | 0.68 | - | - | - | 0.52 | - | 15.40 | 16.61 |
|  | Total (%) | 0.16 | 1.90 | 2.10 | 1.10 | 4.31 | 18.52 | 13.69 | 9.72 | 1.54 | 5.44 | 0.73 | 1.44 | 21.05 | 1.62 | 1.29 | 15.40 | 100.00 |
| Overall accuracy = 91 % | |  |  |  |  |  |  |  |  |  |  |  |  |  |  |  |  |  |
